# Supplementary material for: Brain tumour segmentation with incomplete imaging data
Source: Brain Commun. 2023 Apr 28;5(2):fcad118. doi: 10.1093/braincomms/fcad118 (PMC10144694; doi:10.1093/braincomms/fcad118)
Supplement: fcad118_Supplementary_Data [file fcad118_supplementary_data.zip › Supplementary_figure_legend.docx]

**Supplementary Figure 1: Model architecture.**
